# Validation of the Jefferson Scale of Physician Empathy in Spanish medical students who participated in an Early Clerkship Immersion programme

**DOI:** 10.1186/s12909-018-1309-9

**Published:** 2018-09-12

**Authors:** José M. Blanco, Fernando Caballero, Fernando J. García, Fernando Lorenzo, Diana Monge

**Affiliations:** 1 Valle de la Oliva Healthcare Centre, Enrique Granados 2, 28222, Majadahonda, Madrid, Spain; 2 School of Health Sciences, Universidad Francisco de Vitoria, Ctra. Pozuelo-Majadahonda (M-515) Km. 1.800, 28223, Pozuelo de Alarcón, Madrid, Spain; 3 Applied Epidemiology Department of the National Epidemiology Centre, Instituto de Salud Carlos III, C/Sinesio Delgado, 28029 Madrid, Spain; 4 C/Alimoche 30. Urb. Molino de la Hoz, Las Rozas de Madrid, 28232 Madrid, Spain

**Keywords:** Empathy, Medical students, Validity, Reliability, Medical education

## Abstract

**Background:**

The Jefferson Scale of Physician Empathy is the most widely used instrument to measure empathy in the doctor-patient relationship. This work pursued cultural adaptation and validation of the original scale, in its health professions version (JSE-HP), for medical students who participate in an Early Clerkship Immersion Programme of a Spanish university.

**Methods:**

The questionnaire was replied by 506 1st, 2nd, 3rd and 5th year medical students from Universidad Francisco de Vitoria, Madrid, in 2014 and 2016. Internal consistency was analysed by means of Cronbach’s alpha, and reliability by means of test-retest using the intraclass correlation coefficient and the Bland-Altman method. The construct validity was checked by means of confirmatory factor analysis and association with other empathy-related variables. Criterion validity was compared using Davis’ Interpersonal Reactivity Index.

**Results:**

Cronbach’s alpha was 0.82 (range 0.80–0.85). Item-total score correlations were positive and significant (median 0.45, *p* <  0.01). The test-retest intraclass correlation coefficient was 0.68 (0.42–0.82). The factor analysis confirmed the three original factors: “perspective taking”, “compassionate care” and “standing in the patient’s shoes”. Women and students who preferred specialities focused on persons obtained the best scores. The JSE-HP scores were positively correlated with Interpersonal Reactivity Index, personality traits were associated with empathy, clinical interview skills and Objective Structured Clinical Examinations.

**Conclusion:**

The results support the validity and reliability of JSE-HP applied to Spanish medical students.

**Electronic supplementary material:**

The online version of this article (10.1186/s12909-018-1309-9) contains supplementary material, which is available to authorized users.

## Background

Empathy is a key element in the patient-physician relationship [1]. The effort made by a physician to empathize with patients is an act of ethical nature that concerns the core of clinical care. In addition, it has been related to greater patient [2–4] and provider satisfaction [5–7], and lower rates of professional burnout [8–10], but it has also been associated with physician’s improvement in clinical competence [1], including adherence to treatments [11, 12], and other important outcomes in chronic diseases such as diabetes [13, 14].

Within the framework of patient care, Hojat [15–17] defined empathy as “a predominantly cognitive (not only emotional) attribute which involves an understanding (not only feeling) of the experiences, concerns and perspectives of the patient, combined with a capacity to communicate this understanding”, suggesting that both components of empathy (cognition and emotion) are not completely independent. Different empathy measurement tools have been used [18–21]; however, most of them were not designed in the specific context of the patient-doctor relationship. In 2000, Hojat et al. developed a specific valid and reliable instrument to measure the degree of empathy in medical students: the Jefferson Scale of Physician Empathy [15] in its version for students (JSE-S). Subsequently, it was adapted to be applicable to health professions [16] (JSE-HP) and health provider students (JSE-HPS). Both JSE versions are similar. The one used for medical students (JSE-S) is written in the third person and endeavours to reflect the attitude of students towards empathy in medical care, placing them in a secondary position as observers. The version for health professions (JSE-HP) is written in the first person and values the self-perceived empathic behaviour of the physician during appointments with patients. As the authors of the scale claim, the JSE-HP can be used in students who are already in contact with patients [15, 16]. Hojat et al [17] did not find differences when using JSE-S and JSE-HP in a before-after crossover study with 42 internal medicine residents. The correlations between the scores of the two versions were 0.85 (*p* <  0.01) with no differences in Cronbach’s alpha or significant changes in the ratings of the scale.

Since its creation, the JSE is the most widely used empathy measurement scale in the medical environment. Multiple studies [22–24] have corroborated its validity and reliability, not only with medical students and health professionals, but also with students of other healthcare professions [25]. It has been translated, culturally adapted and validated for more than 56 languages/dialects and has been used in at least 80 different countries [26].

It has been found that women obtain higher scores in JSE [15, 16, 27, 28], as well as students with a preference for studying specialities focused on persons (family medicine, psychiatry, paediatrics, internal medicine, etc.) [28–30]. Other studies have observed that students with better scores in the Objective Structured Clinical Examinations (OSCEs) or rotating internships [31] also obtained significantly higher scores in JSE; these results were not associated with appraisals of their academic knowledge. Positive correlations between scores in JSE and some desirable personality traits [32, 33] such as *agreeableness*, *openness to experience*, *conscientiousness* and *extroversion* have been found. Other projects have studied the differences in JSE scores among students [34] of different university courses.

Some universities have appraised the empathic attitudes and behaviours of their students, and have even compared such attitudes and behaviours [35]. Different studies [36, 37] have described a downward trend in JSE scores during the course of medical studies (especially from the start of the clinical training period) and in subsequent specialisation [38, 39]. Other authors [40, 41] do not agree with this approach and believe that there are sociocultural variables to take into account.

A Best Evidence Medical Education [42, 43] review has reported the benefits of early contact of medical students with real clinical practice. Early contact with patients improves the empathy, communication skills and clinical skills of the students, as well as their motivation, self-confidence, satisfaction and positive attitudes. Contact with real patients help students to contextualise theoretical learning and enhance their vision of psychological, family-related and social aspects of the illness. Furthermore, it allows them to interact with health professionals who are going to be their role models and to analyse the strengths and weaknesses of the health system. In this type of curriculum, the student actively participates in the engagement with patients, and JSE-HP, which is written in the first person, is a good tool to measure their empathy. It would be desirable for all universities to adopt models in which the boundary between the pre-clinical and clinical periods is less marked, as we understand that in the classic curricular designs, JSE-S has a greater relevance in the empathy analysis of students than JSE-HP. For Spanish, there is a JSE-S validation conducted with Mexican medical students [44], but the socio-cultural differences made it necessary for us to translate and adapt the JSE to be a valid and reliable measuring instrument in our environment. In Spain, while this study, which began in 2014, was being conducted, two works of cultural adaptation and validation in our setting with practising health professionals [45] (JSE-HP) and with medical students [46] (JSE-S) had been published. This last study adapted the Mexican version of the JSE-S and not the original in English. The JSE-HP can be used with medical students who have already been in contact with patients, usually in their third year of the degree. Students who have been in contact with patients since the beginning of the degree are able to take the role of a doctor and respond in the first person to the JSE-HP. In Spain, there are no validation studies of the JSE-HP applied to medical students who participate in Early Clerkship Immersion Programmes. This article describes the process of translation, cultural adaptation and validation of JSE-HP for medical students from a Spanish university, analysing their psychometric properties and results.

Of the two JSE versions, we have opted for JSE-HP. Our students participate in a specific Early Clerkship Immersion (ECI), where they come into contact with patients at the beginning of their course. The JSE-HP items, written in the first person, were more akin to our teaching objectives, forcing students to greater identification and involvement with the situation described by the scale.

Validating JSE-HP for Spanish medical students will open the doors to other studies which appraise the trend of their scores longitudinally over time and the correlation between the self-perceived level of empathy and their objective clinical skills. It will also answer the question of whether students with more empathy choose specialities focused on persons or whether it is contact with medical practice in specialised settings what makes levels drop. On the other hand, we will be able to know the impact of different teaching activities which pursue strengthening empathy [47].

## Methods

### Participants

The study was conducted in the private Universidad Francisco de Vitoria (UFV) of Madrid (Spain), with 506 medical students in their 1st, 2nd, 3rd and 5th year. Fourth-year students participated in the pilot study of the apparent and content validity. At the time of the study, the university did not have students yet in their last medical year (the sixth year).

### Measures

The original Jefferson Scale of Empathy (JSE-HP) in English was used. It is made up of 20 items with scoring using a 7-points Likert scale (1 = strongly disagree, 7 = strongly agree). Ten of the 20 questions are valued negatively (and rectified positively in the subsequent analysis), in order to reduce the effect of acquiescence when responding. The range of possible scores goes from 20 to 140 points. The highest scores are associated with a greater degree of empathy. Even though there is no time limit, it is usually answered in less than 5 min. The JSE-HP has three dimensions. Dimension 1 (Perspective Taking) reflects cognitive empathy. Dimension 2 (Compassionate Care) is the emotional empathy. Dimension 3 (Standing in the Patient’s Shoes) makes up a residual dimension.

### Procedures

**Fig. 1:**
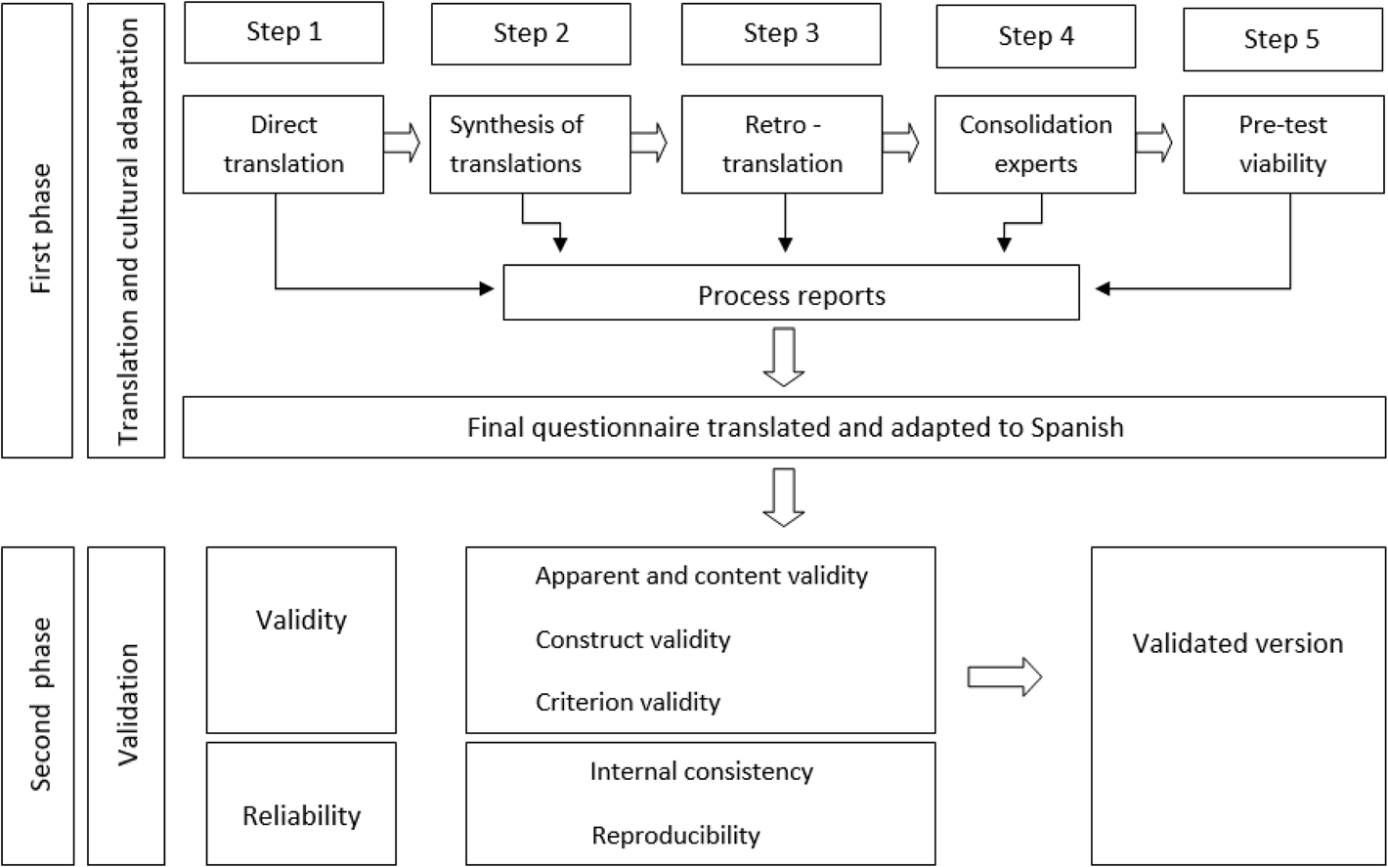
Translation, cultural adaptation and validation algorithm of Spanish JSE-HP

The procedure, in compliance with the description of different authors [48–50], followed two phases (Fig. 1). Firstly, adaptation of the questionnaire to our setting by means of translation - back translation, cultural and linguistic adaptation, analysis of interpretability and ease of understanding. Secondly, check on the validity of content (adjustment of items and dimensions as assessed by experts and students), validity of construct (factor analysis and interrelation with other measurements and known scoring patterns) and criterion validity (association with scores of another empathy scale). Finally, assessment of the reliability of JSE, determining the internal consistency (precision of the instrument based on the uniformity of items in an administration) and the reproducibility of the scale when repeated in time.

In the first phase (translation and cultural adaptation), two bilingual translators with Spanish as their mother tongue (one of them knew the objective of the study) carried out two independent translations, with subsequent consensus summary. Afterwards, two bilingual translators with English as their mother tongue (both were unaware of the objective of the work) conducted a back translation, comparing it with the original version. The final text was consolidated by a committee of 8 physicians who were experts in different departments (communication, ethics, research, medical education) and a linguist.

The text was tested with a sample of 16 4th year medical students. They held a structured interview to analyse possible difficulties in comprehension. No element of the translated and culturally adapted Spanish version of JSE-HP presented difficulties in comprehension in our setting. No inconsistencies in apparent validity or content were detected. The final questionnaire was headed by a note in which students were asked for sincerity and which explained its anonymous and non-academically qualifying nature.

In the second phase (check on the validity and reliability of the JSE), the final version *(*see Additional file 1*)* was answered in hard copy format by 506 students who voluntarily accepted to do so. The participants made up over 90% of the total students enrolled in each year. Randomly, 48 of them repeated the test three months later.

For construct validity, the empathy-related personality traits of VipScan (personality test conducted by students on entering UFV), the performance of students in video-recorded clinical interviews with standardised patients, and the Objective Structured Clinical Examinations (OSCE) of the sample were measured. The overall academic results were also appraised under the assumption that they would not be associated with the overall scores of JSE-HP. The variations in JSE results by gender and preference of the students for a certain medical speciality was analysed, comparing students who had chosen specialities focused on persons that can be practised in an outpatient environment (family medicine, internal medicine, psychiatry and paediatrics) with students centered on technology and procedures (surgery, anatomopathology, radiology, anaesthesia …).

Convergent criterion validity analysed the correlation between JSE and Interpersonal Reactivity Index (IRI) scores.

The participation of the students was voluntary and anonymous. Verbal consent from the students was obtained. At this point we obtained the approval of the Ethics Committee of UFV and of the Postgraduate Research Committee.

### Data analysis

The assumption that the three original factors found in the Jefferson Physician Empathy Scale (JSE-PH) would be reproduced in the sample used for the transcultural adaptation was determined by means of confirmatory factor analysis. We only took the first measurements in all students taking part in the study. The model goodness of fit was measured by means of χ^2^ of the likelihood ratio test, the root mean square error of approximation (RMSEA), the comparative fit index (CFI), the Tucker-Lewis Index (TLI) and the standardised root mean squared residual (SRMR). The reference values used are described in the results section, in addition to the values obtained in the confirmatory factor analysis.

The association between continuous variables and scales was conducted by means of Pearson or Spearman correlations, whichever was applicable.

The internal consistency of the JSE scores was analysed by means of Cronbach’s alpha coefficient and the item-total correlation. The reproducibility was analysed by means of the test-retest intraclass correlation coefficient, the Student’s t-test for paired data and the Bland-Altman method. Acceptable values for the test in the validation and adaptation stage [51–54] were: 0.7 for Cronbach’s alpha coefficient; 0.5 as moderate and 0.7 as high value for the test-retest intra-class correlation coefficient, and a positive and statistically significant correlation for all item-total values.

For statistical analyses, the SPSS Statistics_21 software and STATA, version 14.2, using structural equation models with the “sem” option for the confirmatory factor analysis, were used. An a priori alpha significance level = 0.05 was set for all analyses.

## Results

### Descriptive information

Five hundred and six students made up the study sample, of whom 72.9% were women. Of the total sample, 27% were from the first, 26.6% from the second, 21% from the third and 25.4% from the fifth year, with 72.6%, 67.9%, 76% and 73.6% women, respectively.

Each year, the percentage of replies to the test exceeded 90%. Five questionnaires (1%) were excluded due to failure to fill in more than 20% of the items. There were 39 blank responses from a total of 9880 (0.4%), which were replaced by the mean score obtained by the student in the remaining items of the questionnaire, given that the rate of non-answered items was below 4, as recommended by the authors of the original JSE [15].

The mean score obtained by our students in the JSE-HP was 120.71 points and the standard deviation was 11.48. The other descriptive statistics broken down by genders are shown in Table 1*.* There were no ceiling or floor effects.

**Table 1:** Descriptive statistics of JSE-HP Spanish version applied to a Spanish sample of medical students

Interval	Men *N* sample = 137 (27.1%)			Women. *N* sample = 369 (72.9%)			Total. *N* sample = 506		
	Freq	Aggregate Freq	Range Percentile %	Freq	Aggregate Freq	Range Percentile %	Freq	Aggregate Freq	Range Percentile %
≤80	4	4	1–2	0	0	1	4	4	1
81–85	0	4	1–2	0	0	1	0	4	1
86–90	1	5	3	0	0	1	1	5	1
91–95	3	8	4–5	5	5	1	9	14	1–2
96–100	6	14	6–10	10	15	2–3	16	30	3–5
101–105	13	27	11–17	9	24	4–6	22	52	6–9
106–110	10	37	18–26	11	35	7–9	21	73	10–14
111–115	19	56	27–40	45	80	10–21	65	138	15–27
116–120	18	74	41–52	53	133	22–35	71	209	28–40
121–125	25	99	53–71	72	205	36–54	97	306	41–59
126–130	20	119	72–86	78	283	55–76	99	405	60–79
131–135	13	132	87–96	59	342	77–93	72	477	80–94
> 135	5	137	97–100	17	359	94–100	22	499	95–100
Lost	0	137		5	364		5	506	
Descriptive statistics									
Mean	116.42			122.42			120.71		
Median	119			124			122		
Standard dev	14.19			9.71			11.48		
Possible range	20–140			20–140			20–140		
Actual range	49–140			91–140			49–140		

#### Construct validity: Confirmatory factor analysis

The confirmatory factor analysis checked the association of the three dimensions with all the expected observed variables (*p* <  0.05). Adequate co-variances were also obtained between the three dimensions (between 0.12 and 0.23). In the goodness of fit criteria [55], although the χ^*2*^ value was high, the Chi-squared/degrees of freedom (χ^*2*^/gl) was 1.97, RMSEA was below 0.06 (0.05), the comparative fit index (CFI) was greater than 0.90 (0.93), and Tucker-Lewis Index (TLI) although lower than the optimum value of 0.95, was still high (0.92). The standardised root mean residual (SRMR) was below 0.05 (0.04). In summary, the goodness of fit of the model was considered appropriate, and it did not require any modification Table 2.

**Table 2:** Cut-off Criteria for Several Fit Indexes and the values obtained in a Spanish sample of 506 medical students [58]

Statistics	Abbreviation	Criterion	Results
Absolute adjustment			
Chi-squared/degrees of freedom	χ2/gl	< 3	1.97
Comparative adjustment			
Comparative Fit Index	CFI	≥0.90	0.93
Tucker-Lewis Index	TLI	≥0.95	0.92
Other			
Standardised Root Mean squared Residual	SRMR	< 0.05	0.04
Root mean square error of approximation	RMSEA	< 0.06	0.05

### Construct validity: Association with other empathy-related variables

Women obtained scores 6 (3.41–8.60) points higher than men scores in total JSE-HP score. All dimensions of JSE-HP scored significantly higher in women (Table 3). No significant correlations were found between age and JSE-HP score. Students with a preference for future specialisation centered on persons obtained scores (mean 125.83; standard deviation 8.50) that were higher in JSE-HP (in total and dimension 2: emotional empathy) than the scores of students who opted for specialities focusing on technology or procedure (mean 120.21; standard deviation 10.93) (Table 3*).*

**Table 3:** Scores by gender (*N* sample = 506) and specialisation preference (*N* sample = 91) of JSE-HP Spanish version, in Spanish medical students

	Mean	SD	Mean differences ^a^*p* < 0.01 ^b^*p* < 0.05	Cohen’s value
Dimension 1				
Women	61.62	5.98	2.89^a^(1.34–4.44)	0.40
Men	58.73	8.42		
Dimension 2				
Women	49.01	4.44	2.65^a^(1.52–3.77)	0.50
Men	46.36	6.10		
Dimension 3				
Women	11.81	2.06	0.49^b^(0.08–0.90)	0.23
Men	11.32	2.16		
JSE-HP total				
Women	122.42	9.73	6.00^a^(3.41–8.60)	0.50
Men	116.42	14.19		
Dimension 1				
Person-related	64.00	5.11	2.68(−0.56–5.91)	0.43
Technology-Procedures	61.32	7.21		
Dimension 2				
Person-related	50.17	4.52	2.42^b^(0.16–4.68)	0.52
Technology-Procedures	47.75	4.78		
Dimension 3				
Person-related	11.65	2.06	0.52(−0.57–1.61)	0.24
Technology-Procedures	11.13	2.33		
JSE-HP total				
Person-related	125.83	8.50	5.62^b^(0.64–10.60)	0.57
Technology-Procedures	120.21	10.93		

^a^*p* < 0.01

^b^*p* < 0.05

Significant positive correlations were found between JSE-HP results or any of its dimensions and different VipScan personality traits, such as empathy, responsibility and ability to relate to others, as well as OSCE and clinical interview skills of the students in video-recorded meetings with standard simulated patients*.* The correlation was negative for neuroticism and non-existent for the academic performance of the students *(*Table 4*).*

**Table 4:** Correlations of JSE-HP scores in its Spanish version with other variables for medical students

Variable	*N* sample	Women %	Dimension 1	Dimension 2	Dimension 3	JSE-HP Total ^a^*p* < 0.01 ^b^*p* < 0.05
IRI Perspective taking	188	68.1	0.32^a^	0.12	0.26^a^	0.28^a^
IRI Empathic concern	188	68.1	0.39^a^	0.38^a^	0.22^a^	0.44^a^
IRI Fantasy	188	68.1	0.22^a^	0.22^a^	0.14	0.25^a^
IRI Total score	188	68.1	0.33^a^	0.22^a^	0.18	0.32^a^
VipScan Empathy	95	71.6	0.19	0.17	0.16	0.22^b^
VipScan Responsibility	95	71.6	0.32^a^	0.20	0.27^a^	0.33^a^
VipScan Ability to relate	95	71.6	0.21^b^	−0.03	0.30^a^	0.15
VipScan Neuroticism	95	71.6	−0.21^b^	− 0.12	−0.26^b^	− 0.22^b^
OSCE Anamnesis	89	73	0.23^b^	0.04	−0.10	0.15
OSCE Communication	89	73	0.30^a^	−0.09	0.08	0.17
OSCE overall results	89	73	0.23^b^	−0.02	− 0.10	0.12
Overall rating of VRCI	171	70.2	0.09	0.16^b^	−0.02	0.14
Overall academic mark of the year	191	70.1	0.03	−0.01	− 0.16^b^	−0.03

*JSE-HP* Jefferson Scale of Physician Empathy for health professions, *IRI* Interpersonal Reactivity Index, *OSCE* Objective Structured Clinical Examination. *VRCI* video-recorded clinical interview-

^a^*p* < 0.01

^b^*p* < 0.05

## Convergent criterion validity

A significant positive correlation was found between the JSE-HP scores obtained (in total and by dimensions) and the overall results and those of the IRI sub-scales: perspective taking, empathic concern and fantasy *(*Table 4*)*.

### Reliability: Internal consistency and stability of the results

The mean scores of the items of our JSE-HP version range from 6.4 to 3.83 *(*Table 5*)*. Even if answers were obtained from a wide range of possibilities of the Likert scale, they were asymmetric, with a tendency towards the higher values of the scale. The item with highest mean scores and, in turn, the lowest standard deviation, was item number 2: *“My patients feel better when I understand their feelings”.* Internal consistency is shown in Table 5*.* No item is dispensable as the Cronbach’s alpha obtained of 0.82 would not significantly improve. There is positive and significant correlation between each of the items and the overall result of the scale, the median being 0.45 (*p* <  0.01).

**Table 5:** Reliability of JSE-HP in its Spanish version applied to a Spanish population of medical students

	Mean	Standard deviation	Alpha if item eliminated	Correlation item-total
Item 1	6.46	1.33	0.82	0.30^a^
Item 2	6.49	0.86	0.81	0.56^a^
Item 3	5.49	1.46	0.83	0.23^a^
Item 4	6.17	1.10	0.82	0.45^a^
Item 5	4.97	1.46	0.83	0.14
Item 6	6.18	0.98	0.82	0.42^a^
Item 7	6.46	1.08	0.82	0.45^a^
Item 8	6.37	1.17	0.81	0.48^a^
Item 9	6.22	1.09	0.81	0.55^a^
Item 10	6.12	1.12	0.81	0.58^a^
Item 11	6.27	1.09	0.81	0.53^a^
Item 12	6.00	1.64	0.83	0.28^a^
Item 13	6.14	1.07	0.81	0.60^a^
Item 14	6.48	1.03	0.82	0.41^a^
Item 15	6.16	1.28	0.81	0.50^a^
Item 16	6.26	0.93	0.81	0.68^a^
Item 17	5.84	1.23	0.82	0.44^a^
Item 18	3.83	1.44	0.83	0.14
Item 19	6.35	1.24	0.83	0,15^a^
Item 20	6.43	0.90	0.81	0.59^a^

*JSE-HP* Cronbach’s Alpha: 0.82 Median Correlation item-total: 0.45

^a:^ *P*. 0.01. *N* sample: 506

**Fig. 2:**
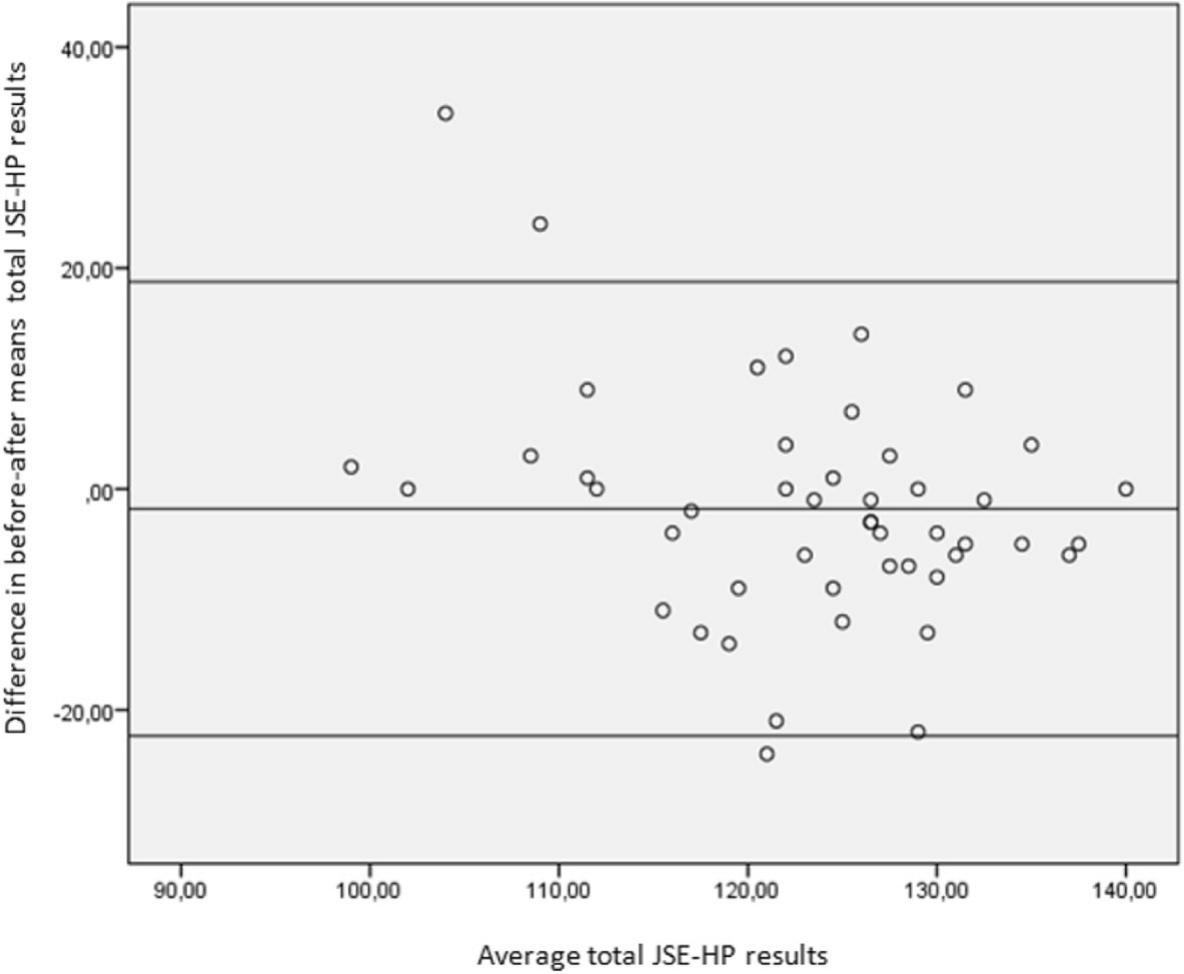
Stability of test re-test response of JSE-HP Spanish version. N = 48

The intraclass correlation coefficient, which measures the stability of the results of the questionnaire in 48 students after three months, was 0.68 (95% *CI*: 0.42–0.82). There were no differences in the before-after means (in total and by dimension) after the Student’s t-analysis for paired data. Average of differences was − 1.83 (95% *CI*:-4.88–1.22). Figure 2 shows the Bland-Altman graphs for the overall JSE-HP results, which represent the degree of agreement of the test-retest. The analysis of posterior linear regression found no variations in the differences in regard to means when analysed by dimensions, although it found significant variations when analysing the overall results of the scale. B -0.35 (95% *CI*:-0.51–0.2). The results by dimensions are available in the Additional files 2, 3 and 4.

## Discussion

The data provided in this work support the validity and reliability of our version of JSE-HP, which is a translated and culturally adapted version to be presented to Spanish medical students. The results confirm the validity of its content, construct and criteria, as well as its consistency and reproducibility.

The percentage of answers to the scale in our study (more than 90% in all the years) was very high, making the sample highly representative. In order to avoid bias in the selection, it was ensured that the percentage of women of the sample was not different from that of the total students of each year.

Similarly to what is described in other countries and to different versions of JSE [31, 27, 28], in our setting, women also obtained significantly higher scores than men, as is the case for the students who preferred specialities centered on persons (family and community medicine, internal medicine, paediatrics and psychiatry) as compared to those who chose specialities focused on technology and procedures.

The significant and positive association between the JSE-HP scores and the personality traits theoretically associated with the concept of empathy of the VipScan tool (empathy, responsibility and ability to relate) are comparable to those described by Hojat [15, 16] et al. in the development of JSE, and this supports the construct validity of the scale. The same occurs with neuroticism, where the correlation was negative.

The association with OSCE ratings and communication skills of students in video-recorded interviews with simulated patients was also positive, although to a lesser degree. These results are to be expected, as these concepts are mutually related, although they are not equivalent.

The associations found with Davis’ Interpersonal Reactivity Index, a measurement scale of empathy in the general population, were similar to those described by Hojat [15] et al. in the scale’s design, which provides criteria validity to our version of JSE-HP.

The internal consistency and stability of the results after repeating the questionnaire were acceptable [51] for this type of test in the field of psychology. Cronbach’s alpha in our analysis (0.82) was similar to the score obtained by Hojat [15] et al. in the original scale, as well as the score obtained by other countries and in our setting [39]. Hojat [16] et al. found a test reliability and confirmation score of 0.65 (*p* < 0.01) after administering the scale again after 3–4 months to 71 professionals, while in our study we obtained a score of 0.68 (0.42–0.82).

Although we could consider this as a limitation of the study, based on the fact that JSE measures empathic attitudes and not real behaviour, it could be assumed that both go hand in hand in order to avoid psychological stress in the individual, a phenomenon known as “cognitive dissonance”. On the other hand, different studies highlighted a relationship between self-perceived empathy by medical students [56] and physicians [57], measured by JSE-HP, and the actual or simulated patient’s perception of empathy analysed by the Jefferson Scale of Patient’s Perceptions of Physician Empathy.

Another limitation is the phenomenon of social desirability, where we have attempted to reduce the effect by means of the mentioned anonymity and confidentiality measures. Hojat [28] et al. studies show the low impact of this phenomenon on the results obtained. In our case, there were no differences in the results obtained by JSE-HP when excluding the students who performed the personal interview, while we detected an excess of “desirable” answers in the VipScan personality analysis.

Another problem arises when generalizing these results. The sample was opportunistic in only one private university in Madrid and it may not represent all medical students in Spain, although the similarities described with the samples obtained by Ferreira-Valiente et al [46] with medical students in two Catalan universities (a state university and a private one) point in the same direction.

The JSE-HP scores obtained by our students reflect moderate-high levels of empathy if compared to those described in other studies [27, 44, 40]. Even if we cannot prove it, it is possible that the admission system for students in our university, which takes into consideration not only academic performance but also desirable personality traits to make a good doctor, supported by the VipScan psycho-technical test and personal interviews, creates a desirable bias in the screening. Just as described by other authors [31], in our study we do not find any association between the empathy levels measured using JSE-HP and the academic performance of the students. This fact opens a door for reflection on how to select the students who can study medicine in the different state and private centres.

For universities, it is of utmost interest to analyse the degree of empathy of students who wish to embark on studies with a high humanistic charge, such as medicine, as well as to monitor it in time and assess the impact of the different educational programmes pursuing to maintain and strengthen said empathy [47].

A BEME [43] (Best Evidence Medical Education) review of 2013 supports the role of “role modelling” and personal reflections, ideally guided by the university, as the most effective in integrating professionalism in medical schools. In Universidad Francisco de Vitoria, the teaching of professionalism is present in the explicit curriculum, reinforced in experiential learning, with a space for reflection on fitting and non-fitting behaviour in this regard in standard clinical practice (concealed curriculum). The early contact of students with clinical reality (Early Clerkship Immersion) favours this learning [42].

Our focus is optimistic. Empathy can be modulated by means of suitable educational programmes. The professors of universities and postgraduates in medicine are mentors or role models and can play a decisive role in improving the empathic skills of students and residents and their capacity to advance together, fostering teamwork, towards the ideal of professionalism.

Future research is required to help us define empathy levels in our environment, which we can classify as deficient, acceptable or excellent, or use ratio percentiles in a similar way as proposed by Hojat [27].

Furthermore, future studies which can fathom the underlying causes of the difference between genders observed in empathy levels are desirable, as well as to know if the more empathic students are those who opt for specialities centered on the person or, on the contrary, whether it is the more technological and procedure-based environments that undermine empathy in the relationship with the patient.

JSE measures the self-perceived empathy of the student. There is a need for further studies of the assessment of student empathy from the perspective of real or simulated patients. Different works have shown the importance of empathy in the patient’s satisfaction [2], the improvement in therapeutic compliance [11] and the health outcomes [13, 14].

## Conclusions

Our work is the first conducted in Spain confirming the psychometric qualities of the Jefferson Scale of Physician Empathy in its version for health professions (JSE-HP) applied to medical students. In our opinion, the Early Clerkship Immersion Programme qualifies students to interact with real and simulated patients and allows them to answer the JSE-HP questionnaire.

The data provided in this work support the validity and reliability of our JSE-HP version used for Spanish medical students.

The results confirm its construct validity based on a three-factor model (perspective taking, compassionate care and standing in the patient’s shoes). The culturally-adapted instrument will allow us to detect the differences between the degree of empathy of medical students in Spain, assess its evolution over time, carry out comparisons among different universities and analyse the impact of different educational programmes which pursue its stimulation.

## Additional files

Additional file 1: JSE-HP Spanish version. Retro-translation JSE-HP Spanish version. The Jefferson Scale of Physician Empathy health professionals version, translated, adapted and validated to Spanish and its retro-translation to English. (DOCX 25 kb)

Additional file 2: Stability of test re-test response of dimension 1 of JSE-HP Spanish version. *N* = 48. Stability of test re-test response of dimension 1 of JSE-HP Spanish version, measured using the Bland-Altman method in 48 medical Spanish students. (DOCX 159 kb)

Additional file 3: Stability of test re-test response of dimension 2 of JSE-HP Spanish version. *N* = 48. Stability of test re-test response of dimension 2 of JSE-HP Spanish version, measured using the Bland-Altman method in 48 medical Spanish students. (DOCX 171 kb)

Additional file 4: Stability of test re-test response of dimension 3 of JSE-HP Spanish version. *N* = 48. Stability of test re-test response of dimension 3 of JSE-HP Spanish version, measured using the Bland-Altman method in 48 medical Spanish students. (DOCX 155 kb)

## Abbreviations

BEME: Best Evidence Medical Education
CFI: Comparative fit index
ECI: Early Clerkship Immersion
IRI: Interpersonal Reactivity Index
JSE: Jefferson Scale of Physician Empathy
JSE-HP: Jefferson Scale of Physician Empathy version for health professions
JSE-HPS: Jefferson Scale of Physician Empathy version for students of health professions
JSE-S: Jefferson Scale of Physician Empathy version for students
OSCE: Objective Structured Clinical Examinations
RMSEA: Root mean square error of approximation
SRMR: Standardised root mean squared residual
TLI: Tucker-Lewis Index
UFV: Universidad Francisco de Vitoria
