# Correction to: Validation of the Jefferson Scale of Physician Empathy in Spanish medical students who participated in an Early Clerkship Immersion programme

**DOI:** 10.1186/s12909-022-03344-4

**Published:** 2022-04-13

**Authors:** José M. Blanco, Fernando Caballero, Fernando J. García, Fernando Lorenzo, Diana Monge

**Affiliations:** 1 Valle de la Oliva Healthcare Centre, Enrique Granados 2, 28222 Majadahonda, Madrid, Spain; 2 School of Health Sciences, Universidad Francisco de Vitoria, Ctra. Pozuelo-Majadahonda (M-515) Km. 1.800, 28223 Pozuelo de Alarcón, Madrid, Spain; 3 Applied Epidemiology Department of the National Epidemiology Centre, Instituto de Salud Carlos III, C/Sinesio Delgado, 28029 Madrid, Spain; 4 C/Alimoche 30. Urb. Molino de la Hoz, 28232 Las Rozas de Madrid, Madrid, Spain

**Correction to: BMC Medical Education 18, 209 (2018)**

**https://doi.org/10.1186/s12909-018-1309-9**

Following publication of the original article [1], the authors became aware that they had not obtained permission from the *Asano-Gonnella Center for Research in Medical Education and Healthcare’* to publish the authors’ Spanish version of the *Jefferson Scale of Physician Empathy* scale (health professionals version).

Additional File 1, which contained a copy of the scale, has therefore been removed from our original article. The translated scale is available with permission from the *Asano-Gonnella Center for Research in Medical Education and Healthcare* by sending an email to: empathy@jefferson.edu

The original article has been corrected.
