# Supplementary material for: Validation of the Jefferson Scale of Physician Empathy in Spanish medical students who participated in an Early Clerkship Immersion programme
Source: BMC Med Educ. 2018 Sep 12;18:209. doi: 10.1186/s12909-018-1309-9 (PMC6134759; doi:10.1186/s12909-018-1309-9)
Supplement: Supplementary file 1 — JSE-HP Spanish version. Retro-translation JSE-HP Spanish version. The Jefferson Scale of Physician Empathy health professionals version, translated, adapted and validated to Spanish and its retro-translation to English. (DOCX 25 kb) [file 12909_2018_1309_MOESM1_ESM.docx]

**Additional file 1**.

JSE-HP health professionals version, adapted to Spanish

| 1. Comprender cómo se sienten mis pacientes y sus familiares no es un factor relevante para el tratamiento médico. | 1 2 3 4 5 6 7 |
| --- | --- |
| 2. Mis pacientes se sienten mejor cuando comprendo sus sentimientos. | 1 2 3 4 5 6 7 |
| 3. Me resulta difícil ver las cosas desde la perspectiva de mis pacientes. | 1 2 3 4 5 6 7 |
| 4. Considero que entender el lenguaje corporal de mis pacientes es tan importante como la comunicación verbal en las relaciones médico-paciente. | 1 2 3 4 5 6 7 |
| 5. Tengo un buen sentido del humor, lo que creo que contribuye a obtener un mejor resultado clínico. | 1 2 3 4 5 6 7 |
| 6. Como todo el mundo es diferente, me resulta casi imposible ver las cosas desde la perspectiva de los pacientes. | 1 2 3 4 5 6 7 |
| 7. Trato de no prestar atención a las emociones de mis pacientes cuando los entrevisto y hago su historia clínica. | 1 2 3 4 5 6 7 |
| 8. Prestar atención a las experiencias personales de mis pacientes es irrelevante para la efectividad del tratamiento. | 1 2 3 4 5 6 7 |
| 9. Trato de ponerme en el lugar de mis pacientes cuando les atiendo. | 1 2 3 4 5 6 7 |
| 10. El hecho de comprender los sentimientos de mis pacientes les aporta una sensación de reconocimiento que resulta terapéutica por sí misma | 1 2 3 4 5 6 7 |
| 11. Las enfermedades de los pacientes sólo se pueden curar mediante tratamiento médico y, por tanto, ningún vínculo afectivo que establezca  con mis pacientes podrá tener un valor significativo en ese cometido. | 1 2 3 4 5 6 7 |
| 12. Considero que preguntar a mis pacientes sobre lo que sucede en sus vidas es un factor sin importancia para comprender sus quejas físicas. | 1 2 3 4 5 6 7 |
| 13. Trato de comprender qué pasa por la mente de mis pacientes prestando atención a su comunicación no verbal y a su lenguaje corporal. | 1 2 3 4 5 6 7 |
| 14. Creo que no hay lugar para las emociones en el tratamiento de las enfermedades médicas. | 1 2 3 4 5 6 7 |
| 15. La empatía es una destreza terapéutica sin la cual mi éxito como médico estaría limitado. | 1 2 3 4 5 6 7 |
| 16. Un componente importante de la relación con mis pacientes es mi comprensión de su estado emocional y el de sus familiares. | 1 2 3 4 5 6 7 |
| 17. Trato de pensar como mis pacientes para prestarles una mejor atención. | 1 2 3 4 5 6 7 |
| 18. No permito que me afecten las relaciones emocionales intensas entre mis pacientes y sus familiares. | 1 2 3 4 5 6 7 |
| 19. No disfruto leyendo literatura no médica ni con actividades artísticas. | 1 2 3 4 5 6 7 |
| 20. Creo que la empatía es un factor terapéutico importante en el tratamiento médico. | 1 2 3 4 5 6 7 |

© Thomas Jefferson University. All rights reserved.

Retro – translation JSE-HP health professionals version, adapted to Spanish

| 1. My understanding of how my patients and their relatives feel is an irrelevant factor to medical treatment. | 1 2 3 4 5 6 7 |
| --- | --- |
| 2. My patients feel better when I understand their feelings. | 1 2 3 4 5 6 7 |
| 3. I find it difficult to see things from my patients’ perspective. | 1 2 3 4 5 6 7 |
| 4. I believe that understanding my patients’ body language is as important as verbal communication in doctor-patient relationships. | 1 2 3 4 5 6 7 |
| 5. I have a good sense of humour, an aspect I think contributes to obtaining a better clinical outcome. | 1 2 3 4 5 6 7 |
| 6. Since everyone is different, I find it almost impossible to see things from my patients’ perspective. | 1 2 3 4 5 6 7 |
| 7. I try not to pay attention to my patients’ emotions when I interview them and write down their medical record. | 1 2 3 4 5 6 7 |
| 8. Paying attention to my patients’ personal experiences is irrelevant to treatment effectiveness. | 1 2 3 4 5 6 7 |
| 9. I try to put myself in my patients’ shoes when seeing them. | 1 2 3 4 5 6 7 |
| 10. Understanding my patients’ feelings gives them a sense of validation that is therapeutic in itself. | 1 2 3 4 5 6 7 |
| 11. Patients’ illnesses can only be cured by medical treatment and, therefore, no emotional bond established with my patients will have any significant value in that task. | 1 2 3 4 5 6 7 |
| 12. I believe that asking my patients about what is going on in their lives is an unimportant factor in understanding their physical complaints. | 1 2 3 4 5 6 7 |
| 13. I try to understand what is going on in my patients’ mind by paying attention their non-verbal communication and body language. | 1 2 3 4 5 6 7 |
| 14. I don’t think there is any room for emotions in the treatment of medical illnesses. | 1 2 3 4 5 6 7 |
| 15. Empathy is a therapeutic skill without which my success as a doctor would be limited. | 1 2 3 4 5 6 7 |
| 16. An important component of the relationship with my patients is my understanding of their emotional state and that of their relatives. | 1 2 3 4 5 6 7 |
| 17. I try to think like my patients to give them better care. | 1 2 3 4 5 6 7 |
| 18. I don’t let intense emotional relations between my patients and their relatives affect me. | 1 2 3 4 5 6 7 |
| 19. I don’t enjoy reading literature that is not medical or with experiences that are not related to art. | 1 2 3 4 5 6 7 |
| 20. I believe empathy is an important therapeutic factor in medical treatment. | 1 2 3 4 5 6 7 |

© Thomas Jefferson University. All rights reserved.
