# Supplementary figures and images for: Validation of the Jefferson Scale of Physician Empathy in Spanish medical students who participated in an Early Clerkship Immersion programme
Source: BMC Med Educ. 2018 Sep 12;18:209. doi: 10.1186/s12909-018-1309-9 (PMC6134759; doi:10.1186/s12909-018-1309-9)

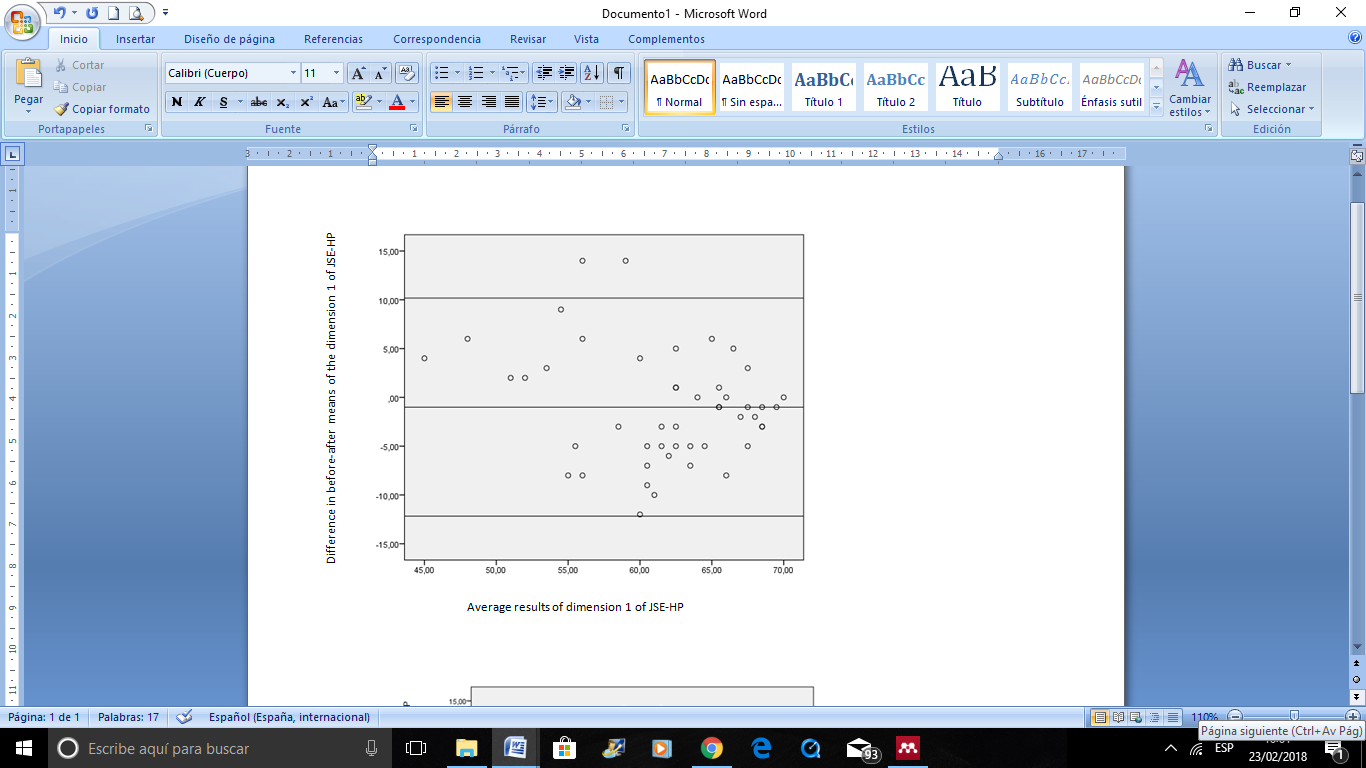

Supplement: Supplementary file 2 — Stability of test re-test response of dimension 1 of JSE-HP Spanish version. N = 48. Stability of test re-test response of dimension 1 of JSE-HP Spanish version, measured using the Bland-Altman method in 48 medical Spanish students. (DOCX 159 kb) [file 12909_2018_1309_MOESM2_ESM.docx]

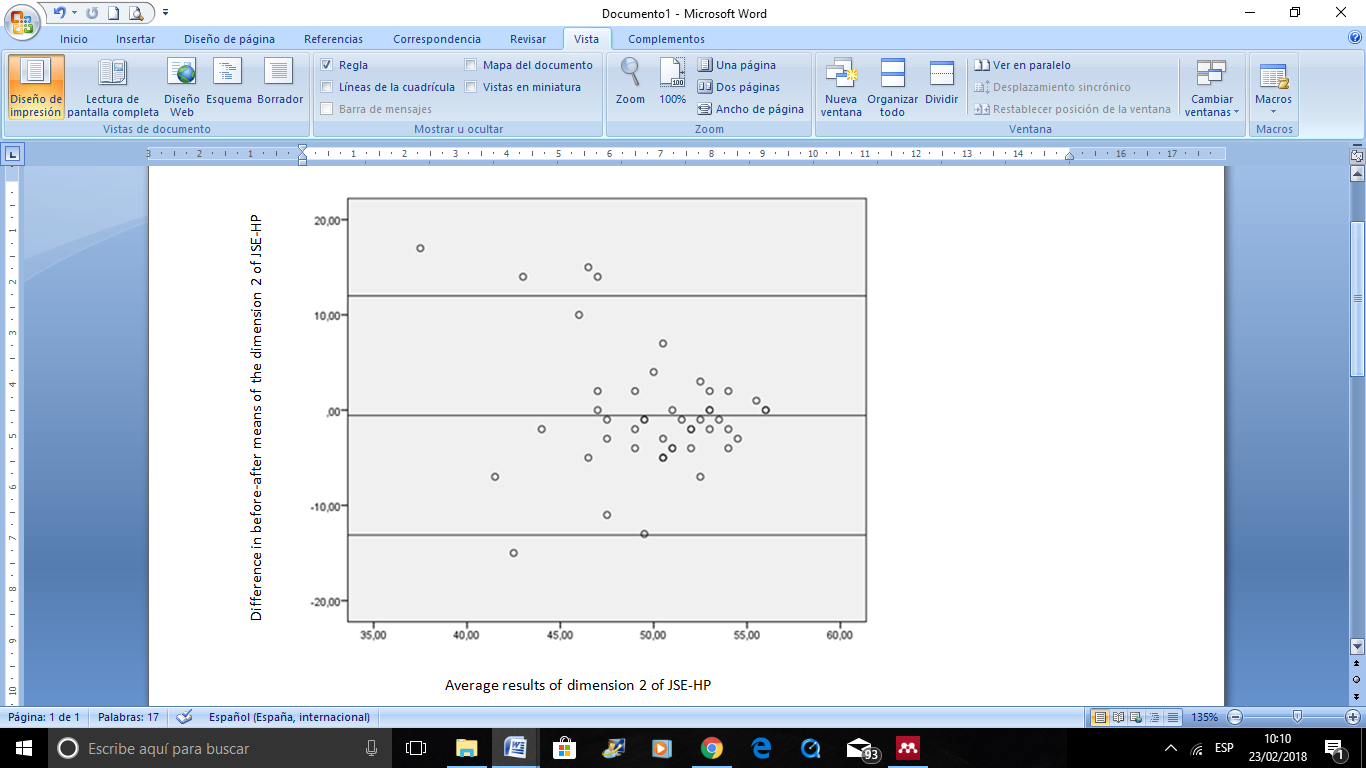

Supplement: Supplementary file 3 — Stability of test re-test response of dimension 2 of JSE-HP Spanish version. N = 48. Stability of test re-test response of dimension 2 of JSE-HP Spanish version, measured using the Bland-Altman method in 48 medical Spanish students. (DOCX 171 kb) [file 12909_2018_1309_MOESM3_ESM.docx]

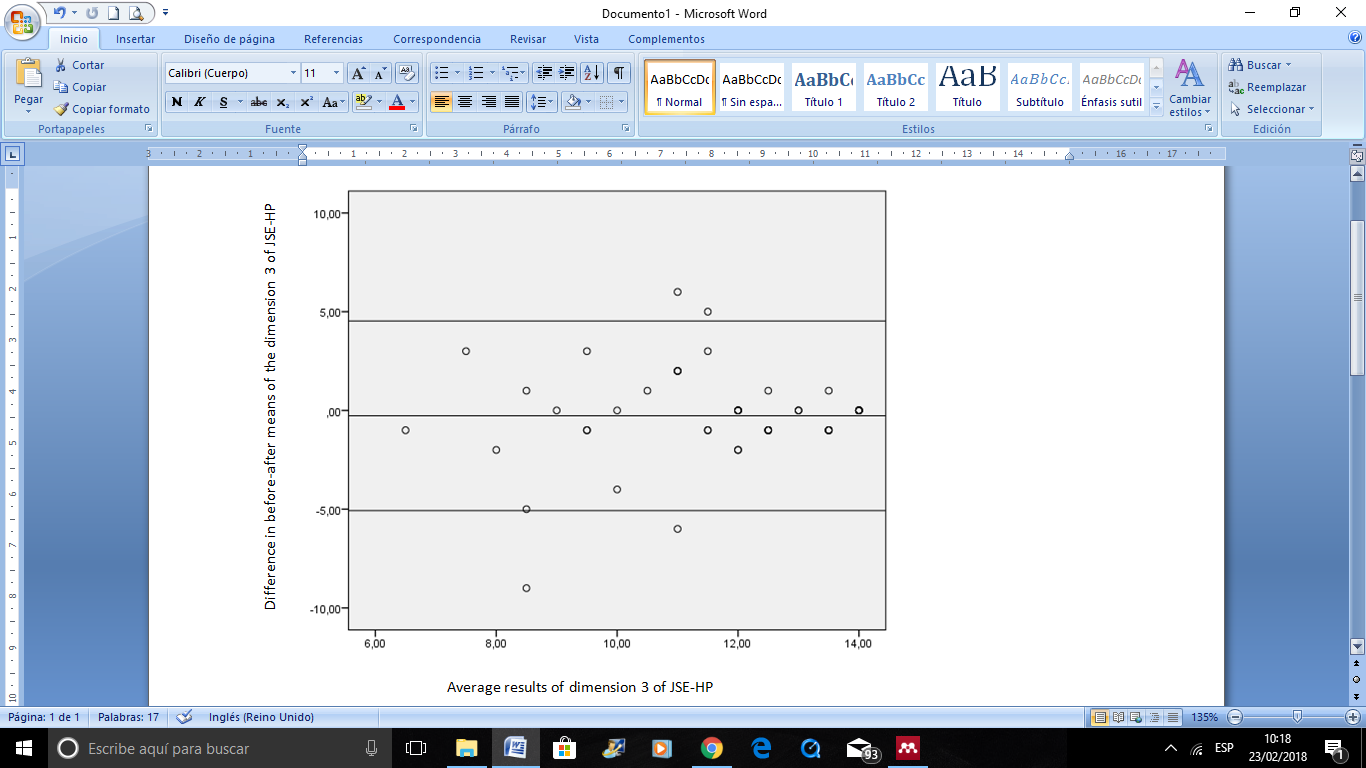

Supplement: Supplementary file 4 — Stability of test re-test response of dimension 3 of JSE-HP Spanish version. N = 48. Stability of test re-test response of dimension 3 of JSE-HP Spanish version, measured using the Bland-Altman method in 48 medical Spanish students. (DOCX 155 kb) [file 12909_2018_1309_MOESM4_ESM.docx]
